# Integrated single-cell and multi-cohort analyses reveal CTCF as a key regulator of chemo-immunotherapy resistance in lung adenocarcinoma

**DOI:** 10.3389/fcell.2026.1922944

**Published:** 2026-09-21

**Authors:** Hui Chen, Taiyang Zhang, Yiwei Zhang, Mohan Li, Haoran Yang, Yue Zhang, Feng Xu

**Affiliations:** 1 Department of Lung Cancer, Tianjin Medical University Cancer Institute and Hospital, National Clinical Research Center for Cancer, Tianjin, China; 2 Tianjin Medical University, Tianjin, China; 3 Department of Pathology, Tianjin Medical University Cancer Institute and Hospital, National Clinical Research Center for Cancer, Tianjin’s Clinical Research Center for Cancer, Tianjin, China

**Keywords:** CD276, chemo-immunotherapy resistance, CTCF, immune microenvironment, non-small cell lung cancer, tumor cell state

## Abstract

**Background:**

Chemo-immunotherapy has improved clinical outcomes in non-small cell lung cancer (NSCLC), but resistance remains a major challenge. The tumor cell states and molecular mechanisms underlying treatment resistance are not fully understood.

**Methods:**

Single-cell RNA sequencing data from NSCLC patients receiving chemo-immunotherapy were integrated with public transcriptomic cohorts and functional experiments. Resistance-associated tumor cell populations were identified, and key regulatory factors were investigated through bioinformatic analyses and experimental validation.

**Results:**

Single-cell analysis identified a malignant epithelial cell subpopulation enriched in patients with stable disease, suggesting a resistance-associated tumor cell state. Genes derived from this population were associated with unfavorable prognosis and were used to construct a prognostic model with consistent performance across multiple independent cohorts. Among the candidate genes, CTCF was identified as a key regulator associated with poor survival and reduced immune infiltration. In an orthotopic lung tumor model, Ctcf knockdown enhanced the antitumor efficacy of pemetrexed combined with anti-PD-1 treatment and promoted CD8^+^ T-cell activity. Mechanistically, CTCF directly upregulated RRM2 and CD276, which mediated chemotherapy resistance and immune suppression, respectively.

**Conclusion:**

Our findings identify CTCF as a critical regulator of chemo-immunotherapy resistance in NSCLC. The CTCF–RRM2/CD276 axis links tumor-intrinsic drug resistance with immune evasion and may represent a potential therapeutic target.

## 1 Introduction

Immune checkpoint inhibitors combined with chemotherapy have improved the treatment of non-small cell lung cancer (NSCLC), particularly in neoadjuvant and perioperative settings. Trials such as CheckMate 816, KEYNOTE-671 and CheckMate 77T showed that chemo-immunotherapy could improve pathological response and survival outcomes compared with chemotherapy alone (Awad et al., 2025; Wakelee et al., 2023; Casc et al., 2024).

Previous studies have mainly attributed immunotherapy resistance to changes in the tumor microenvironment, including T-cell dysfunction, impaired antigen presentation and immunosuppressive cellular components. Recently, single-cell and multi-omic studies have suggested that tumor-cell-intrinsic states also contribute to resistance by shaping immune evasion and therapeutic response (Barkley et al., 2022; Yan et al., 2025). Nevertheless, the key malignant cell states and regulatory factors driving chemo-immunotherapy resistance in NSCLC remain unclear.

CTCF is a chromatin-associated transcriptional regulator involved in genome organization and gene expression control (Ong and Corces, 2014). Although aberrant CTCF binding has been linked to oncogenic transcriptional dysregulation (Fang et al., 2020), its role in NSCLC chemo-immunotherapy resistance is poorly understood. In this study, we integrated single-cell RNA sequencing, public transcriptomic cohorts and functional experiments to identify a resistance-associated malignant epithelial cell state and investigate the role of CTCF in regulating chemoresistance and immune suppression.

## 2 Methods

### 2.1 Study design and data collection

This study integrated single-cell RNA sequencing (scRNA-seq) data, public lung adenocarcinoma transcriptomic cohorts, clinical survival information, and *in vitro* and *in vivo* experimental data to investigate tumor cell states associated with chemo-immunotherapy resistance and their key regulatory factors. The scRNA-seq data were obtained from lung adenocarcinoma patients who received chemo-immunotherapy. Treatment responses were evaluated according to the Response Evaluation Criteria in Solid Tumors (RECIST) (Leong, 2024), and patients were classified into the stable disease (SD) group and the partial response/complete response (PR/CR) group. Public transcriptomic datasets included the TCGA-LUAD cohort and six GEO lung adenocarcinoma cohorts, including GSE13213, GSE26939, GSE29016, GSE30219, GSE31210, and GSE42127. All bioinformatic analyses were performed using R software version 4.1.1.

### 2.2 Processing of single-cell RNA sequencing data and identification of malignant cells

The scRNA-seq data were processed using the Seurat package version 4.0.4. After quality control, normalization, identification of highly variable genes, batch-effect correction, dimensionality reduction, clustering, and UMAP visualization, cell types were annotated according to canonical lineage marker genes. Epithelial cells were then extracted for further analysis. To distinguish malignant tumor cells from normal epithelial cells, genome-wide copy number variation (CNV) inference was performed using the fastCNV package, with endothelial cells designated as the reference (non-malignant) population, given their transcriptional stability and karyotypic normality, which minimizes the risk of misclassification arising from potential malignant contamination within normal epithelial reference populations. Epithelial cells with a CNV score exceeding the mean +2 standard deviations (SD) of the reference endothelial cells were classified as malignant. Canonical epithelial markers (EPCAM, KRT18, KRT19) were further employed to confirm epithelial lineage identity and exclude contamination from non-epithelial or ambiguous cell populations. Malignant epithelial cells satisfying both criteria were retained and re-clustered, and treatment resistance-associated tumor cell subpopulations were identified according to the distribution of cells derived from the SD and PR/CR groups.

### 2.3 Identification and validation of resistance-associated genes

Marker genes of the resistance-associated tumor cell subpopulation were identified and further validated in the TCGA-LUAD cohort and six GEO lung adenocarcinoma cohorts. Public transcriptomic data were subjected to gene annotation, normalization, and batch-effect correction before downstream analyses. The expression levels of candidate genes were compared between lung adenocarcinoma tissues and normal lung tissues. Functional enrichment analyses, including Gene Ontology (GO) and Kyoto Encyclopedia of Genes and Genomes (KEGG) analyses, were performed to characterize the biological functions and pathways associated with these genes. Copy number variation patterns of candidate genes and their associations with overall survival were further evaluated in the TCGA-LUAD cohort.

### 2.4 Development and validation of the prognostic model

A prognostic risk model was constructed using resistance-associated candidate genes through an integrated machine-learning strategy. The algorithms included random survival forest (RSF), Lasso, Ridge, CoxBoost, StepCox, gradient boosting machine (GBM), survival support vector machine (survivalSVM), supervised principal components (SuperPC), partial least squares regression for Cox models (plsRcox), and Elastic Net. The TCGA-LUAD cohort was used as the training cohort, whereas the six GEO cohorts were used as external validation cohorts. The optimal model was selected according to the average concordance index (C-index). Patients were divided into high- and low-risk groups based on the median risk score. Kaplan–Meier survival analysis, time-dependent receiver operating characteristic (ROC) analysis, and principal component analysis (PCA) were performed to evaluate the prognostic performance of the model.

### 2.5 Immune microenvironment analysis

The relationship between the risk score and the tumor immune microenvironment was evaluated using multiple computational approaches. Immunotherapy-related scores were analyzed using The Cancer Immunome Atlas (TCIA) (Long et al., 2025). Tumor purity, immune score, stromal score, and ESTIMATE score were calculated using the ESTIMATE algorithm. Immune cell infiltration was further assessed using TIMER, CIBERSORT, QUANTISEQ, MCP-counter, xCell, and EPIC algorithms. These analyses were used to characterize the association between the prognostic risk score and immune-related features in lung adenocarcinoma.

### 2.6 Clinical relevance of CTCF in lung adenocarcinoma

Core candidate molecules were screened by integrating the prognostic model, univariate Cox regression analysis, single-cell expression patterns, and patient survival analyses. CTCF expression levels and risk scores were extracted from the TCGA-LUAD cohort, and Pearson correlation analysis was performed to evaluate their association. Patients were divided into high- and low-CTCF-expression groups according to the median CTCF expression level. Kaplan–Meier survival analyses were performed in the TCGA-LUAD, GSE13213, and GSE26939 cohorts. The expression distribution of CTCF across different cell types and tumor cells from different treatment-response groups was further examined using the scRNA-seq dataset.

### 2.7 Cell culture and generation of CTCF overexpression and knockdown models

Human lung adenocarcinoma A549 cells and murine Lewis lung carcinoma (LLC) cells were used for *in vitro* and *in vivo* experiments. Cells were cultured in medium supplemented with 10% fetal bovine serum and 1% penicillin–streptomycin at 37 °C in a humidified incubator containing 5% CO_2_. A549 cells were used to establish a CTCF overexpression model, with empty vector-transfected cells serving as controls. LLC cells were used to establish a Ctcf knockdown model, with scramble sequence-transfected cells serving as negative controls. The efficiency of CTCF overexpression or knockdown was confirmed by quantitative real-time PCR (qRT-PCR) and Western blotting. LLC cells used for animal experiments were stably transduced with luciferase.

### 2.8 Orthotopic lung tumor model and flow cytometric analysis

Luciferase-labeled LLC-Scramble and LLC-Ctcf-KD cells were used to establish orthotopic lung tumor models in 6-week-old female C57BL/6 mice. After intrapulmonary implantation of tumor cells, mice were randomly assigned to receive vehicle or pemetrexed combined with anti-PD-1 antibody treatment. Tumor burden was monitored by *in vivo* bioluminescence imaging during treatment, and tumor inhibition rates were calculated based on fluorescence intensity. At the experimental endpoint, tumor tissues were collected and dissociated into single-cell suspensions. Flow cytometry was performed to assess the proportions of CD8^+^ T cells, PD-1^+^TIM-3^+^ exhausted CD8^+^ T cells, Ki-67^+^CD8^+^ T cells, and IFN-γ^+^CD8^+^ T cells, as well as myeloid populations consisting of CD11b^+^Gr-1^+^ myeloid-derived suppressor cells (MDSCs), CD11c^+^ macrophages and CD11b^+^F4/80^+^ macrophages. All animal experiments were reviewed and approved by the Institutional Animal Care and Use Committee (IACUC) of the Institute of Biomedical Engineering, Chinese Academy of Medical Sciences & Peking Union Medical College (Approval No.: BME-IACUC-251226–03).

### 2.9 Statistical analysis

All statistical analyses were performed using R software version 4.1.1. Comparisons between two groups were performed using two-tailed Student’s t-tests or Wilcoxon rank-sum tests, as appropriate. Comparisons among multiple groups were performed using one-way analysis of variance (ANOVA) or Kruskal–Wallis tests. Survival analyses were conducted using the Kaplan–Meier method, and differences between groups were assessed using the log-rank test. Cox proportional hazards regression models were used to calculate hazard ratios (HRs) and 95% confidence intervals (CIs). Correlations were evaluated using Pearson correlation analysis. A P value <0.05 was considered statistically significant.

## 3 Results

### 3.1 Single-cell transcriptomic analysis identifies a chemo-immunotherapy resistance-associated tumor cell subpopulation

To investigate tumor cell states associated with differential responses to chemo-immunotherapy in lung adenocarcinoma, we first performed an integrated analysis of single-cell transcriptomic data. After data integration, all cells were grouped into 16 transcriptionally distinct clusters (clusters 0–15) in the UMAP space. Cells derived from different patients showed substantial overlap without obvious sample-specific clustering, indicating effective integration and batch correction (Figures 1A,B).

**FIGURE 1:**
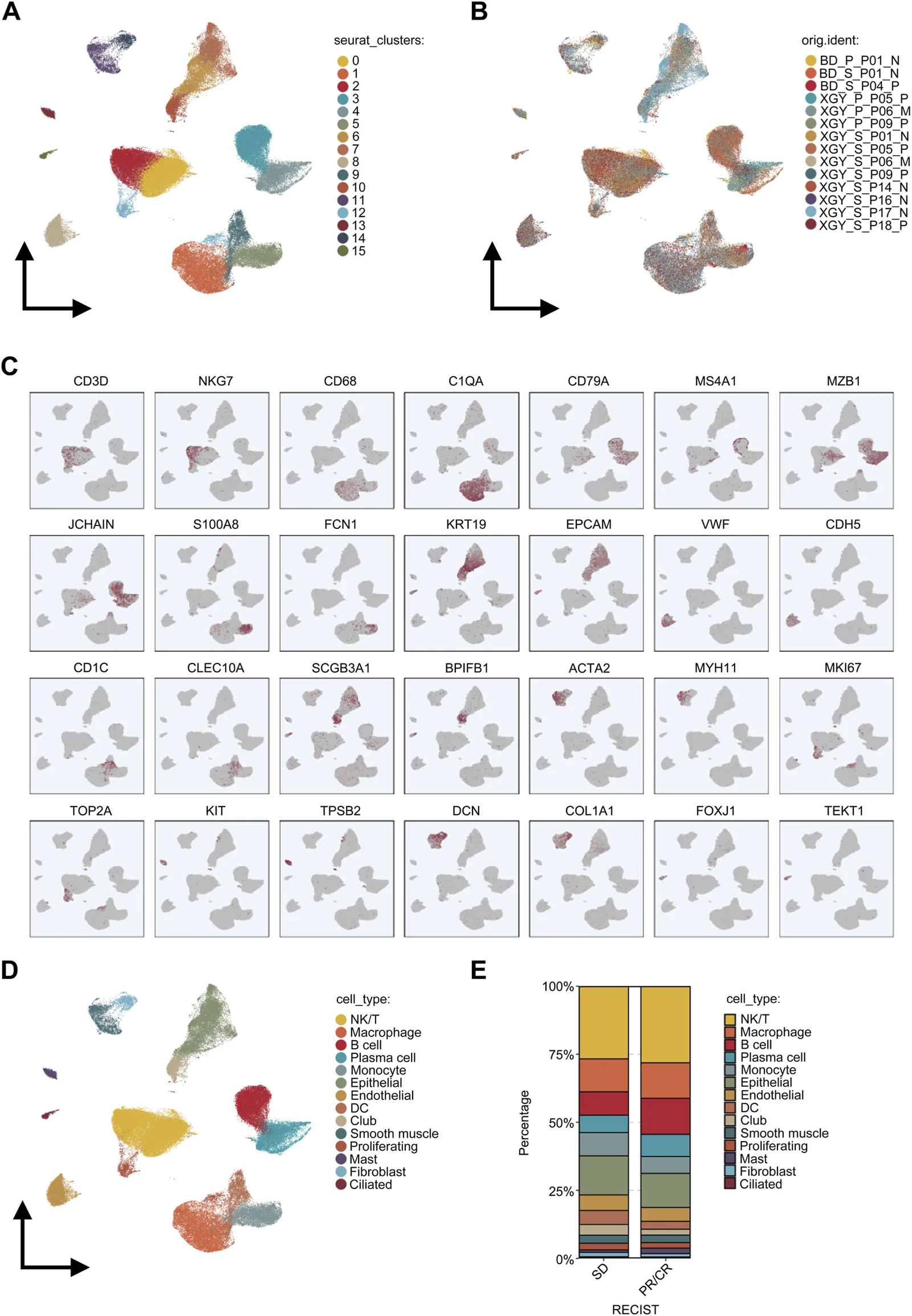
Single-cell transcriptomic landscape of the tumor microenvironment in lung adenocarcinoma patients receiving chemo-immunotherapy. **(A)** After integration of single-cell transcriptomic data from all samples, the unsupervised clustering results of all cells were displayed using UMAP dimensionality reduction. A total of 16 cell clusters were identified (clusters 0–15), with different colors representing different cell clusters. **(B)** In the same UMAP embedding, cells were colored by sample origin, showing the distribution of cells from different patients and tissue-type samples (14 samples in total), reflecting the degree of cell mixing among samples after batch-effect correction. **(C)** Expression distribution of known cell-type marker genes on the UMAP. The depth of red represents the level of gene expression, and gray indicates cells with low or no expression. The displayed marker genes include T/NK cells (CD3D, NKG7), macrophages (CD68, C1QA), B cells (CD79A, MS4A1), plasma cells (MZB1, JCHAIN), monocytes (S100A8, FCN1), epithelial cells (KRT19, EPCAM), endothelial cells (VWF, CDH5), dendritic cells (CD1C, CLEC10A), Club cells (SCGB3A1, BPIFB1), smooth muscle cells (ACTA2, MYH11), proliferating cells (MKI67, TOP2A), mast cells (KIT, TPSB2), fibroblasts (DCN, COL1A1), and ciliated cells (FOXJ1, TEKT1). **(D)** After cell-type annotation based on marker gene expression patterns, the final cell-type identities of all cells were displayed by UMAP. A total of 15 major cell types were identified, with different colors representing different cell types. **(E)** Patients were divided into the stable disease group (SD) and objective response group (PR/CR) according to RECIST response evaluation criteria. The stacked bar plot shows the relative infiltration proportions of each cell type in the tumor microenvironment of the two groups, revealing differences in immune and stromal cell composition between different response groups.

Cell-type annotation based on canonical lineage markers identified 15 major cell populations (Figures 1C,D). Given the focus of this study on tumor-intrinsic mechanisms of chemo-immunotherapy resistance, epithelial cells were selected for subsequent analyses. The cellular composition differed between patients with stable disease (SD) and those with partial or complete response (PR/CR), suggesting that distinct tumor cell states may be associated with treatment response (Figure 1E).

Genome-wide CNV inference was then applied to epithelial cells using endothelial cells as the reference population. This analysis classified epithelial cells into nine CNV clusters. Cluster 5 showed the lowest CNV score and was therefore regarded as normal epithelial cells, whereas clusters 1–4 and 6–9 displayed higher CNV signals and were defined as malignant tumor cells (Figures 2A,B).

**FIGURE 2:**
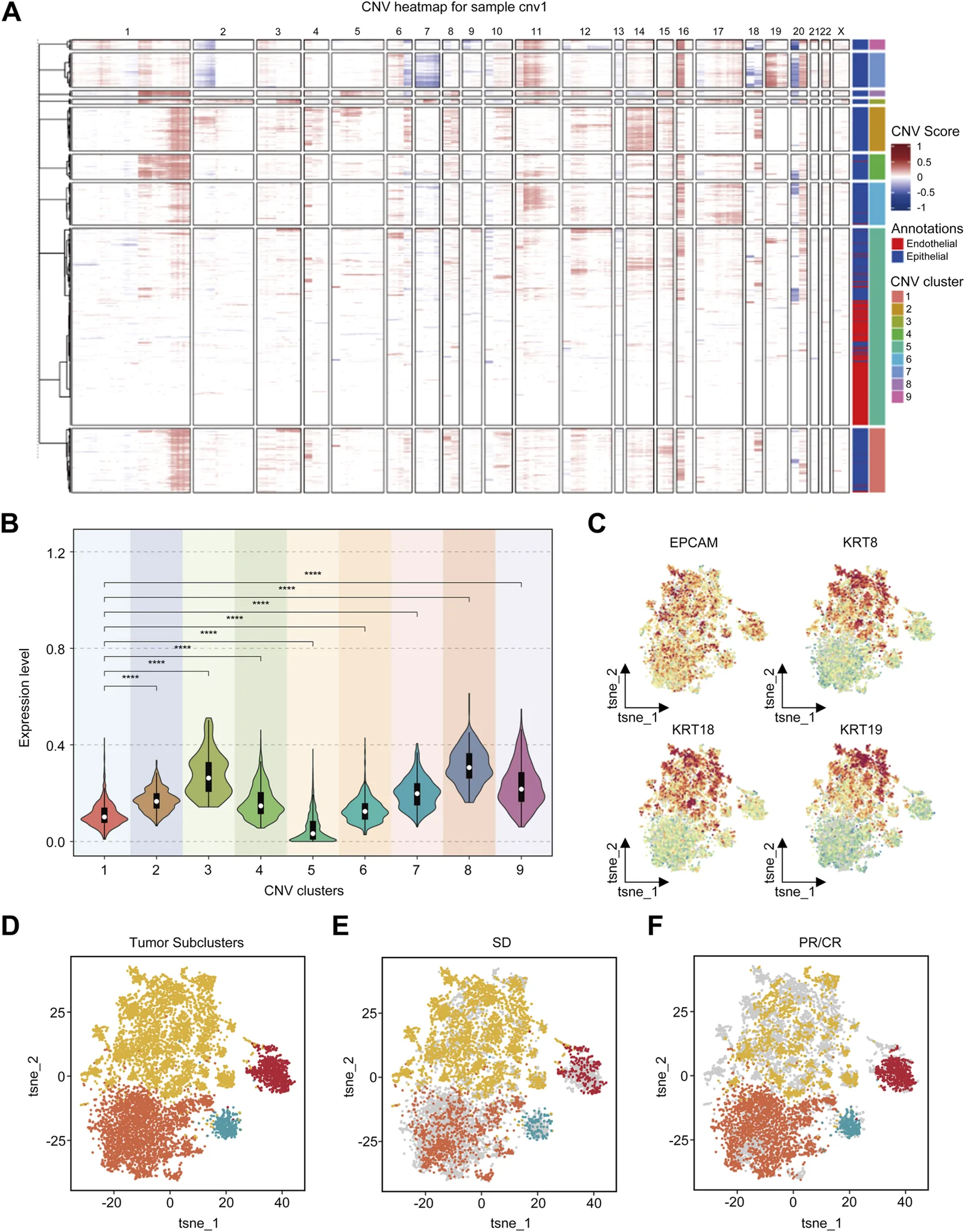
Identification and subclustering of tumor cells. **(A)** Endothelial cells were used as the reference cell population to perform inferCNV copy number variation (CNV) inference analysis on epithelial cells. The heatmap shows the CNV scores of each cell across the whole genome (chromosomes 1–22 and chromosome X), with red indicating copy number amplification and blue indicating copy number loss. The annotation bars on the right indicate cell type (endothelial cells/epithelial cells) and CNV cluster assignment (9 clusters in total). **(B)** Violin plot of the integrated copy number variation scores of each CNV cluster. Cluster 5 showed a significantly lower CNV level (***P < 0.0001 compared with all other clusters), suggesting that this cell population had a relatively stable genome and was defined as normal epithelial cells; the remaining clusters (1–4 and 6–9) showed obvious chromosomal copy number abnormalities and were classified as malignant tumor cells, which were extracted for subsequent analysis. **(C)** Expression distribution of typical epithelial/tumor marker genes (EPCAM, KRT8, KRT18, KRT19) in tumor cells on the t-SNE dimensionality reduction plot. Colors from blue to red represent expression levels from low to high, validating the tumor epithelial identity of the extracted cell population. **(D)** t-SNE visualization of all tumor cells after Seurat reclustering. A total of 4 subclusters were identified (clusters 0–3), with different colors indicating different subclusters. **(E,F)** Subcluster distribution of tumor cells derived from patients in the SD group **(E)** and PR/CR group **(F)** on the t-SNE plot. Colored dots represent tumor cells from each group, and gray dots represent cells from the other group, intuitively showing the differences in tumor cell subcluster composition between the two groups.

Malignant cells expressed canonical epithelial markers, including EPCAM, KRT8, KRT18, and KRT19, confirming their epithelial origin (Figure 2C). Re-clustering analysis identified four malignant tumor cell subpopulations (clusters 0–3) (Figure 2D). Notably, tumor cells from SD patients were predominantly enriched in cluster 0, whereas tumor cells from PR/CR patients were mainly distributed in cluster 1 (Figures 2E,F). To further characterize the functional identity of these subpopulations, GSVA of Hallmark pathways revealed that cluster 0 was preferentially enriched in TGF-β signaling, WNT/β-catenin signaling, and hypoxia-related pathways, suggesting an EMT-like phenotype with a hypoxic component, whereas cluster 1 showed relatively higher activity of proliferation- and DNA repair-related pathways (Supplementary Figure S1A). Therefore, cluster 0 was defined as a chemo-immunotherapy resistance-associated tumor cell subpopulation and selected for subsequent analyses.

### 3.2 Identification and prognostic characterization of resistance-associated tumor-cell genes

To further characterize the molecular features of the chemo-immunotherapy resistance-associated malignant cell population identified by single-cell analysis, we extracted the corresponding tumor cell–derived marker genes and evaluated their expression patterns in bulk LUAD cohorts. After integration of TCGA and GEO datasets, PCA analysis showed no obvious cohort-specific separation, indicating that batch effects were effectively minimized (Figure 3A). Compared with normal lung tissues, tumor samples displayed markedly altered expression patterns of these resistance-associated genes (Figure 3B).

**FIGURE 3:**
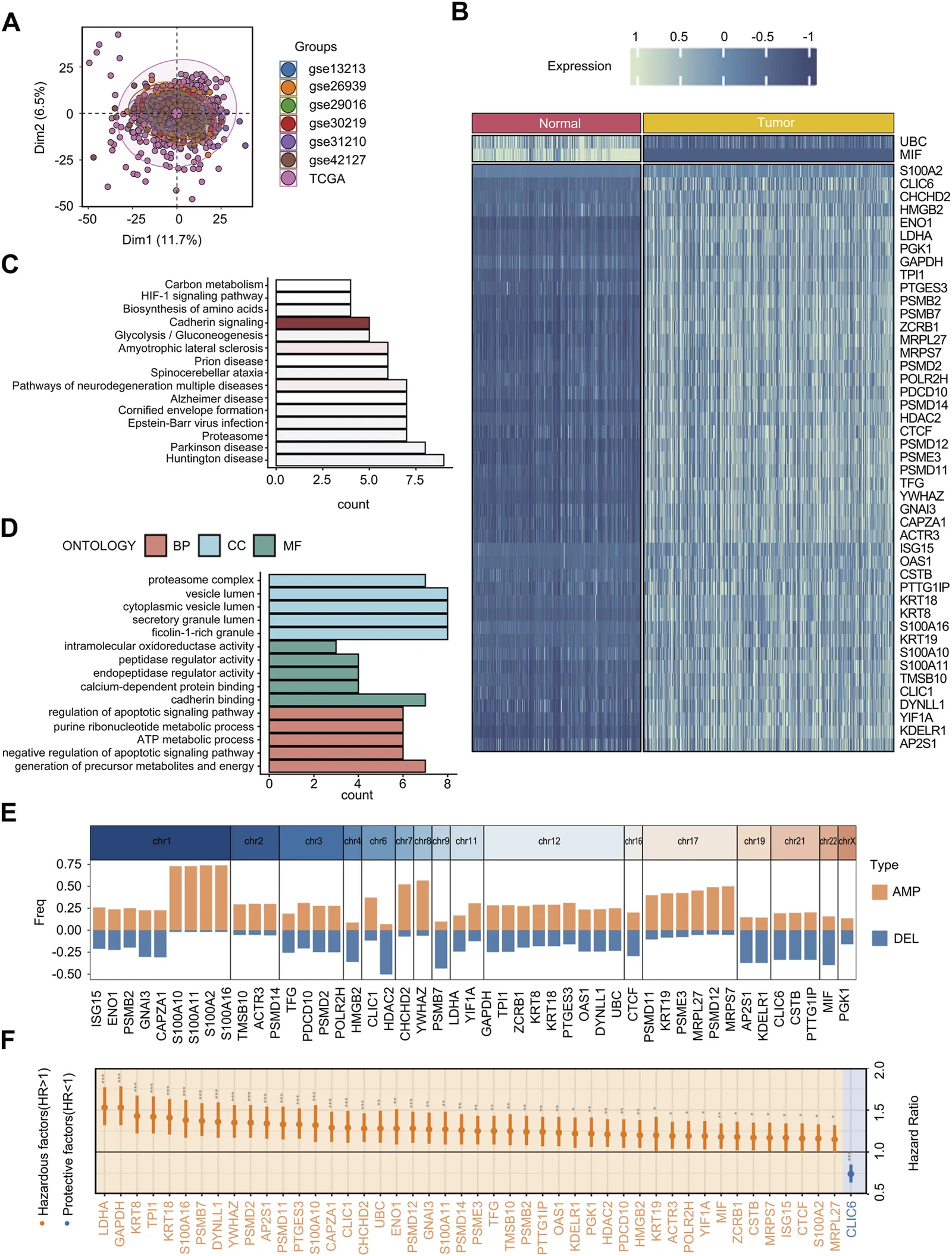
Multi-omics characterization of marker genes in the resistant tumor subcluster (Cluster 0). **(A)** Principal component analysis (PCA) scatter plot after batch-effect correction of seven datasets, including six GEO public datasets (GSE13213, GSE26939, GSE29016, GSE30219, GSE31210, GSE42127) and the TCGA lung adenocarcinoma cohort. **(B)** Heatmap showing the differential expression of Cluster 0 marker genes between normal lung tissues (GTEx) and lung adenocarcinoma tumor samples (TCGA). Rows represent genes, columns represent samples, and colors from blue to yellow indicate expression levels from low to high. **(C)** Bar plot of KEGG pathway enrichment analysis of differentially expressed genes. **(D)** Bar plot of GO enrichment analysis of differentially expressed genes. **(E)** Copy number variation (CNV) frequency profile of differentially expressed genes. Genes are arranged according to their chromosomal locations. Orange bars represent amplification frequency (AMP), and blue bars represent deletion frequency (DEL). **(F)** Univariate Cox proportional hazards regression forest plot based on the TCGA lung adenocarcinoma cohort, showing the hazard ratio (HR) and 95% confidence interval of each differentially expressed gene. Orange dots represent risk factors (HR > 1), and blue dots represent protective factors (HR < 1).

Functional enrichment analysis showed that these genes were mainly involved in tumor-related metabolic and regulatory pathways, including carbon metabolism, HIF-1 signaling, glycolysis/gluconeogenesis, proteasome-related biological processes, ATP metabolic processes, and epithelial–mesenchymal transition-related functions (Figures 3C,D). Copy number variation analysis further revealed frequent genomic alterations among these genes, including both amplification and deletion events across multiple chromosomes (Figure 3E). Univariate Cox regression analysis demonstrated that most genes were associated with increased prognostic risk, whereas CLIC6 acted as a protective factor (Figure 3F). These findings indicate that resistance-associated tumor-cell genes are transcriptionally dysregulated, genomically altered, and clinically relevant in LUAD.

### 3.3 Construction and validation of a machine learning-based prognostic model

To establish a robust prognostic model based on resistance-associated genes, 101 machine-learning algorithm combinations were systematically evaluated across the training and validation cohorts. Among these models, RSF + SuperPC achieved the highest average C-index and was therefore selected as the optimal model (Figure 4A). Based on the calculated risk score, patients were divided into high- and low-risk groups.

**FIGURE 4:**
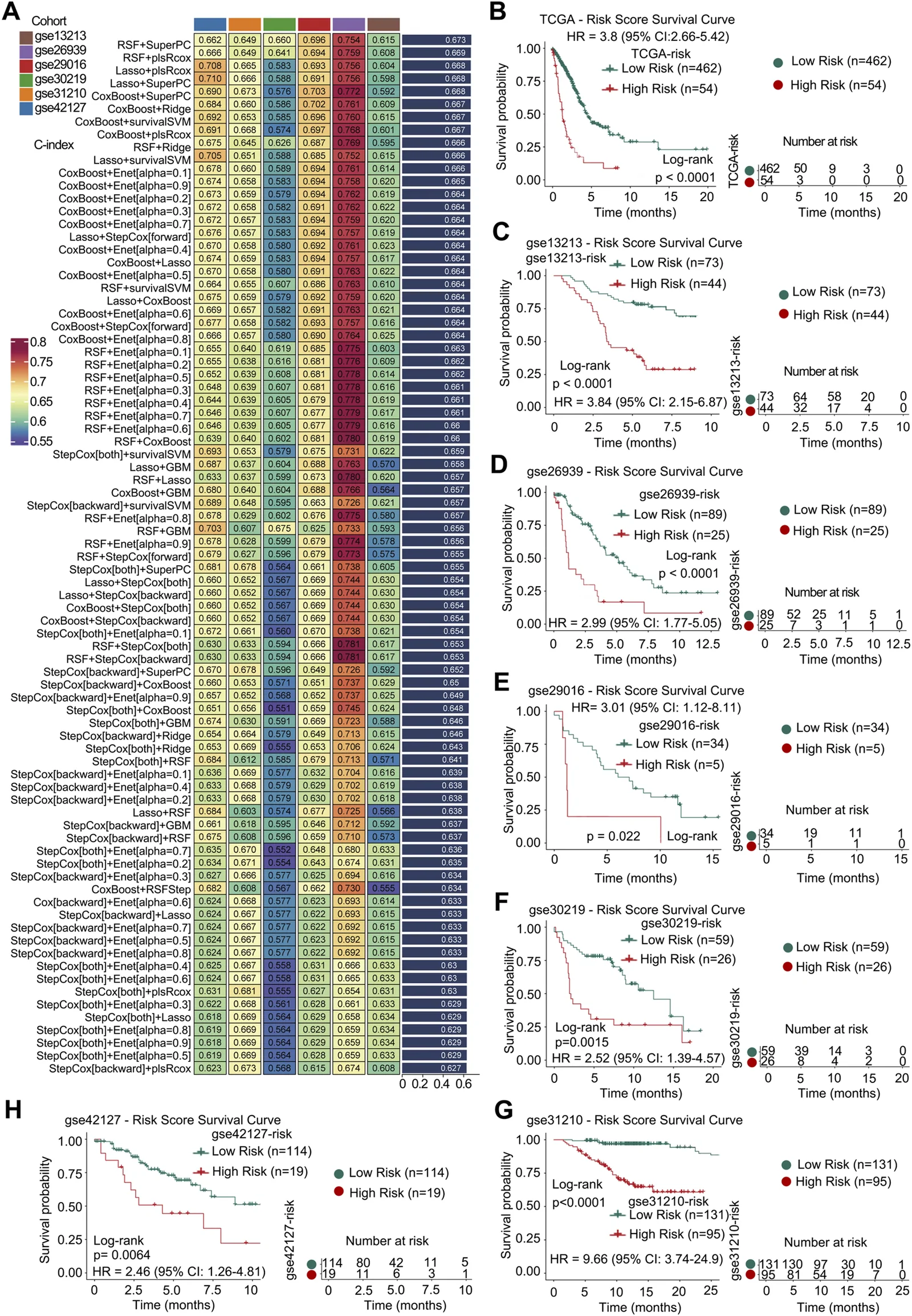
Construction and multi-cohort validation of a machine learning-based prognostic risk model. **(A)** Prognosis-related genes identified in Figure 3 were subjected to pairwise combinations of 10 machine learning algorithms (RSF, Lasso, Ridge, CoxBoost, StepCox [forward/backward/both], GBM, survivalSVM, SuperPC, plsRcox, and Enet [alpha = 0.1–0.9]), generating multiple candidate models. The TCGA cohort was used as the training set, while six independent GEO cohorts (GSE13213, GSE26939, GSE29016, GSE30219, GSE31210, and GSE42127) were used as validation sets. The concordance index (C-index) was calculated for each validation cohort, and the average C-index across the six validation cohorts was used as the criterion for selecting the optimal model. The heatmap shows the C-index values of different models across cohorts, with colors ranging from blue to red, indicating low to high C-index values. The optimal model combination with the highest average C-index (RSF + SuperPC, average C-index = 0.673) was selected for subsequent analyses. **(B–H)** Patients in the TCGA cohort and six GEO validation cohorts were stratified into high-risk and low-risk groups according to the risk score calculated by the optimal model. Kaplan–Meier survival curves show the differences in overall survival between the high-risk group (red) and low-risk group (green) in each cohort. Survival differences were evaluated using the log-rank test. Hazard ratios (HRs) and 95% confidence intervals (CIs) were calculated using Cox proportional hazards models. The numbers of patients at risk at each time point are shown below the curves.

Kaplan–Meier survival analysis showed that patients in the high-risk group had significantly worse overall survival than those in the low-risk group in the TCGA cohort and in multiple independent validation cohorts, including GSE13213, GSE26939, GSE29016, GSE30219, GSE31210, and GSE42127 (Figures 4B–H). In the TCGA training cohort, time-dependent ROC analysis showed that the risk model achieved AUC values of 0.684, 0.678, and 0.645 at 1, 3, and 5 years, respectively; PCA analysis also revealed a clear separation tendency between high- and low-risk patients, supporting the discriminative ability of the risk score in the training dataset (Supplementary Figures S2A,B). Consistently, time-dependent ROC analyses in the external validation cohorts further confirmed the predictive performance of the model, with favorable AUC values at 1, 3, and 5 years in most datasets (Figures 5A–F). In addition, PCA based on the risk model showed clear separation tendencies between the high- and low-risk groups across validation cohorts, suggesting that the model could distinguish patients with different molecular risk states (Figures 5G–L). Together, these results demonstrate that the resistance-associated gene signature has stable and reproducible prognostic value in LUAD.

**FIGURE 5:**
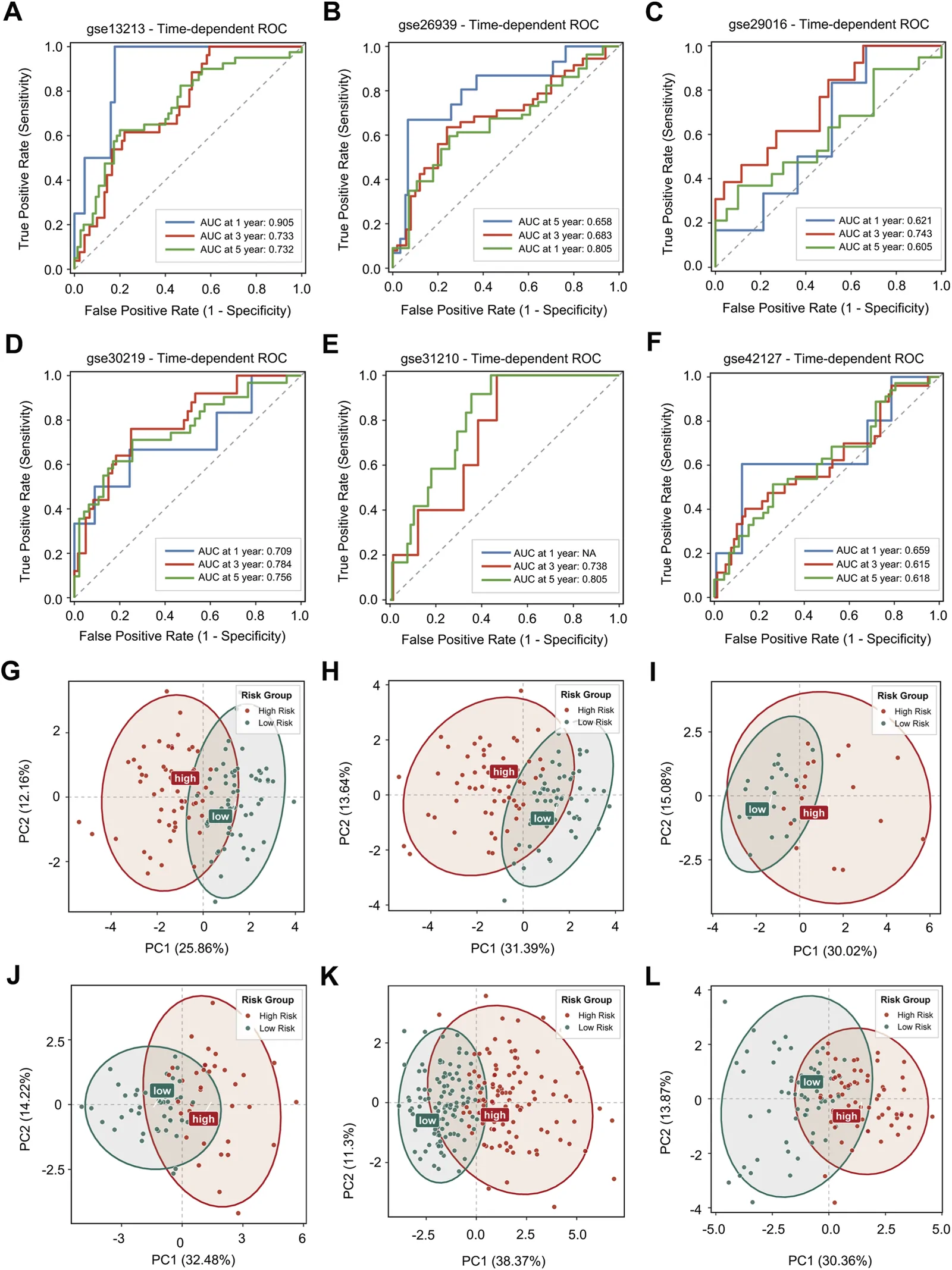
ROC curve analysis and principal component analysis of the risk score model in six GEO validation cohorts. **(A–F)** Time-dependent ROC curves of the risk scores generated by the optimal RSF + SuperPC model in six independent GEO validation cohorts (GSE13213, GSE26939, GSE29016, GSE30219, GSE31210, and GSE42127). The AUC values for 1-year, 3-year, and 5-year survival prediction are shown by the blue, red, and green curves, respectively. **(G–L)** Principal component analysis (PCA) scatter plots of high-risk (red) and low-risk (green) patients based on the expression profiles of model genes in the six GEO validation cohorts.

### 3.4 Association of the risk model with the immune microenvironment and identification of CTCF as a key candidate gene

We next investigated the relationship between the risk score and predicted response to immune checkpoint blockade using the Immunophenoscore (IPS) from The Cancer Immunome Atlas (TCIA). Across all four checkpoint-blockade scenarios—no blockade (CTLA4^−^PD1^-^), anti-CTLA-4 alone (CTLA4^+^PD1^-^), anti-PD-1 alone (CTLA4^−^PD1^+^), and combined anti-CTLA-4 plus anti-PD-1 blockade (CTLA4^+^PD1^+^)—the low-risk group consistently exhibited significantly higher IPS values than the high-risk group (*P* < 0.01 for all comparisons, with the no-blockade and anti-CTLA-4-alone scenarios reaching *P* < 0.001; Figure 6A). These results indicate that low-risk tumors possess greater immunogenicity and a higher predicted likelihood of benefiting from immune checkpoint inhibitors, whether administered as monotherapy or in combination, whereas high-risk tumors displayed consistently lower IPS scores across all scenarios, suggesting reduced predicted responsiveness to checkpoint blockade and a greater propensity toward immunotherapy resistance. Correlation analysis revealed that the risk score was positively correlated with tumor purity, but negatively correlated with StromalScore, ImmuneScore, and ESTIMATEScore (Figures 6B–E). Consistently, immune infiltration analysis using TIMER, CIBERSORT, CIBERSORT-ABS, QUANTISEQ, MCP-counter, xCell, and EPIC demonstrated broad differences in immune cell abundance between the two risk groups (Figure 6F). These results suggest that high-risk tumors are characterized by higher tumor purity and reduced immune/stromal infiltration, whereas low-risk tumors exhibit a more immune-active microenvironment.

**FIGURE 6:**
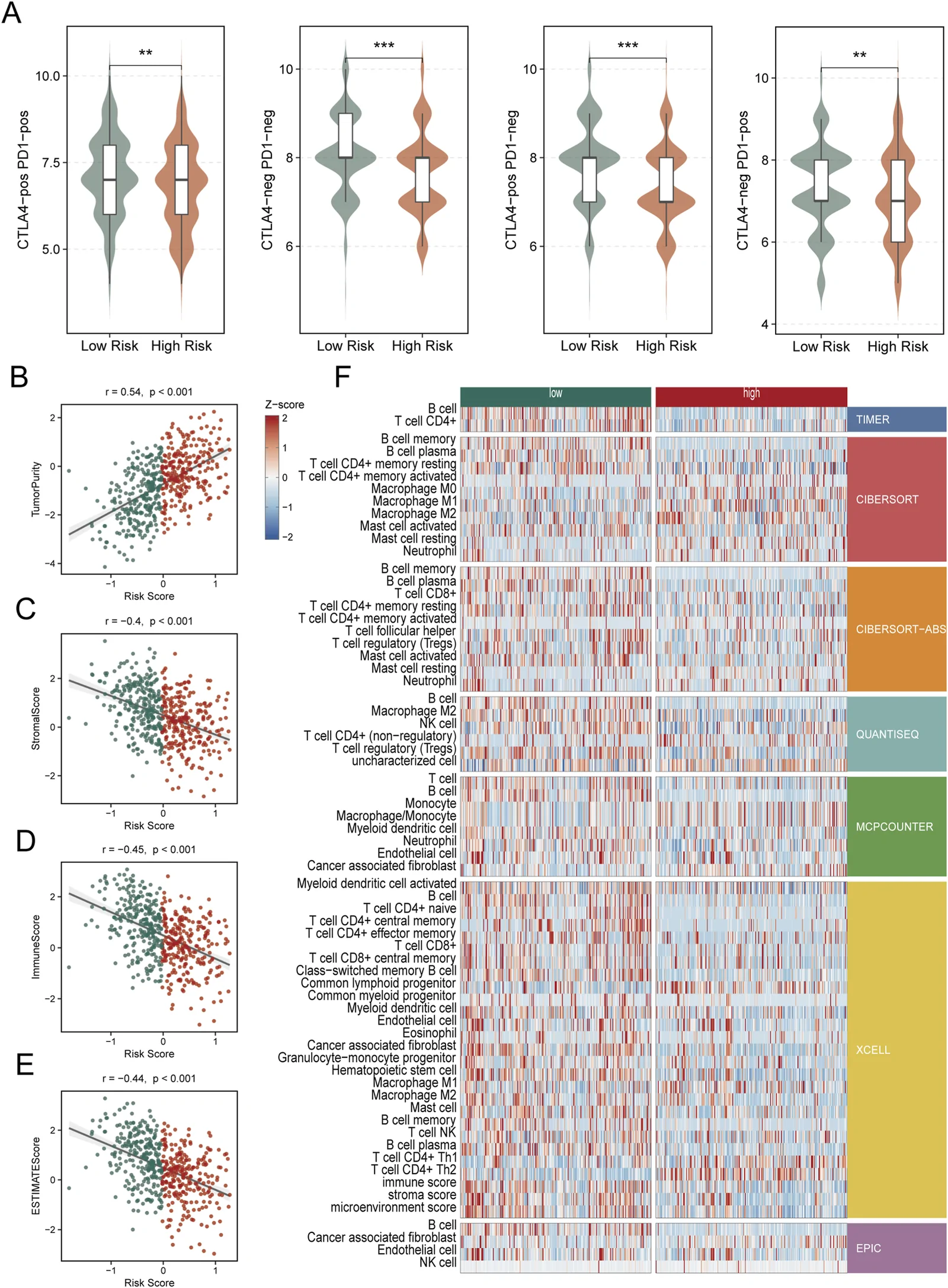
Association between the risk score and the tumor immune microenvironment. **(A)** Based on the TCIA database, differences in four immunophenoscores between the high-risk and low-risk groups were compared. Violin plots show the distribution of immunophenoscores corresponding to the CTLA4^+^PD1^+^, CTLA4^−^PD1^-^, CTLA4^+^PD1^-^, and CTLA4^−^PD1^+^ phenotypes in the two groups. Higher scores indicate a greater potential response to immune checkpoint blockade therapy. **(B–E)** Pearson correlation analyses between the risk score and tumor purity (TumorPurity) **(B)**, stromal score (StromalScore) **(C)**, immune score (ImmuneScore) **(D)**, and ESTIMATE score (ESTIMATEScore) **(E)** calculated by the ESTIMATE algorithm in the TCGA lung adenocarcinoma cohort. Red and green dots represent the high-risk and low-risk groups, respectively. The gray shaded area indicates the 95% confidence interval. Correlation coefficients (r) and corresponding *P* values are shown at the top of each plot (all *P* < 0.001). **(F)** Comprehensive analysis of tumor immune cell infiltration profiles in the high-risk and low-risk groups using seven immune infiltration estimation algorithms, including TIMER, CIBERSORT, CIBERSORT-ABS, QUANTISEQ, MCPCOUNTER, XCELL, and EPIC.

Among the model-related genes, CTCF was further prioritized for downstream validation. CTCF expression was positively correlated with the risk score (r = 0.34, *P* < 0.001; Figure 7A). Survival analysis showed that high CTCF expression was associated with poorer overall survival in TCGA and GSE13213 cohorts, with a similar trend observed in GSE26939 (Figures 7B–D). Single-cell analysis revealed that CTCF was broadly expressed across multiple cell types, with relatively higher expression in epithelial and proliferating cells (Figure 7E). Moreover, CTCF expression was enriched in tumor cells from SD patients compared with PR/CR patients, supporting its potential involvement in chemo-immunotherapy resistance (Figure 7F). These findings identify CTCF as a key candidate molecule linking the resistance-associated risk model with poor prognosis and an immunosuppressive tumor microenvironment.

**FIGURE 7:**
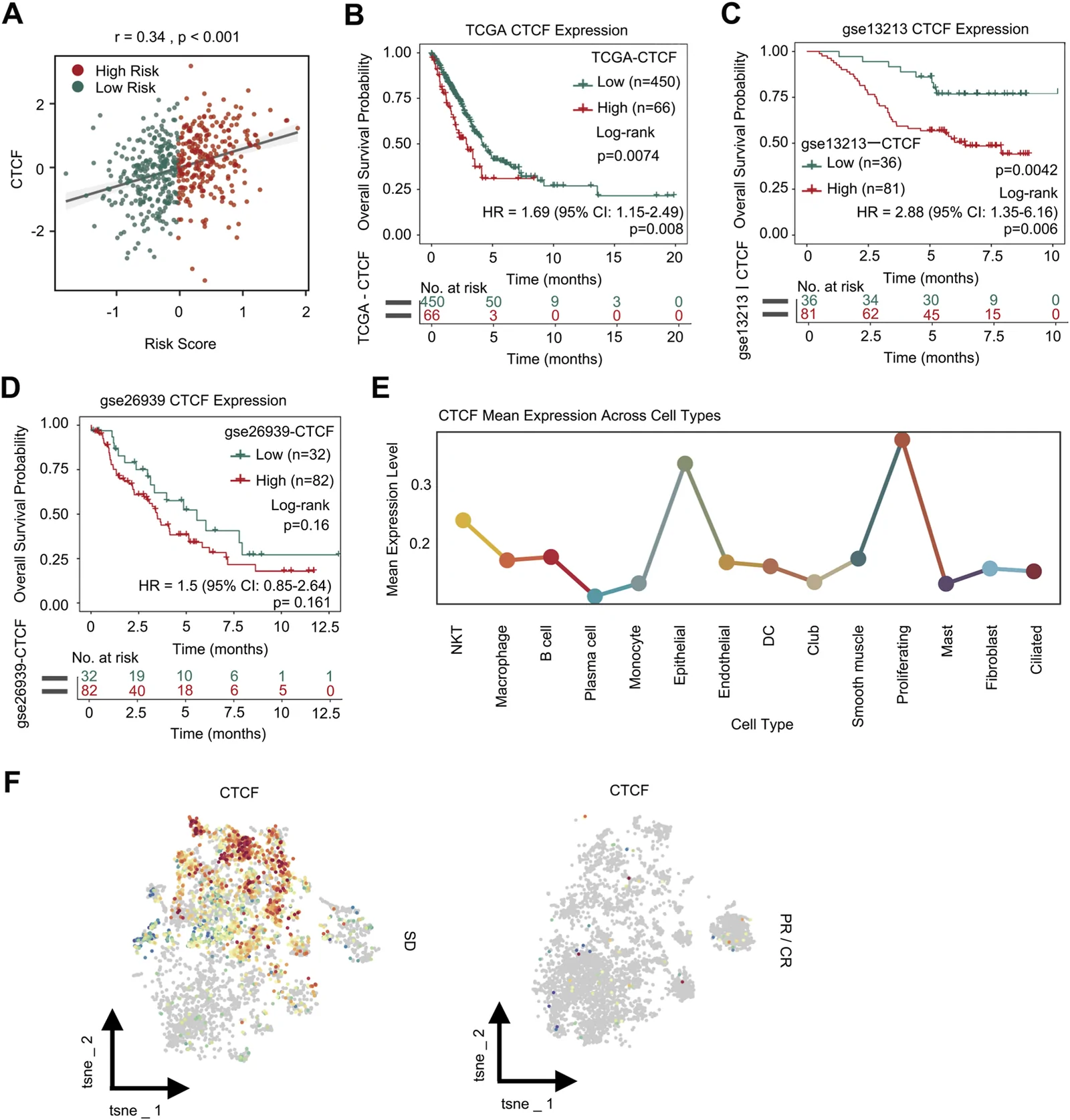
Prognostic value and single-cell expression characteristics of CTCF, the core gene in the risk model. **(A)** In the TCGA lung adenocarcinoma cohort, CTCF expression was significantly positively correlated with the risk score (Pearson r = 0.34, *P* < 0.001). Red and green dots represent patients in the high-risk and low-risk groups, respectively. **(B–D)** Kaplan–Meier survival curves of patients stratified into high- and low-CTCF expression groups based on median expression levels in the TCGA **(B)**, GSE13213 **(C)**, and GSE26939 **(D)** cohorts. **(E)** Line plot showing the average expression of CTCF across 14 cell types based on single-cell transcriptomic data from this study. CTCF was most highly expressed in epithelial cells and proliferating cells, suggesting potential functional activity in tumor cells. **(F)** t-SNE plot showing CTCF expression in tumor cells. Compared with the PR/CR group, CTCF expression was upregulated in tumor cells from the SD group.

### 3.5 CTCF mediates resistance to chemo-immunotherapy in vivo

To validate CTCF as a key molecule in resistance to chemo-immunotherapy for lung adenocarcinoma, we established Scramble and Ctcf-KD cell lines in Luc-labeled LLC cells. Using these 2 cell lines, we constructed orthotopic lung transplantation tumor models in C57BL/6 mice and administered either control treatment or pemetrexed combined with anti-PD-1 chemo-immunotherapy (Figure 8A). The results showed that tumor growth was inhibited in the Ctcf-KD group (Figures 8B,C), accompanied by increased intratumoral CD8^+^ T-cell infiltration and proliferation, as indicated by a higher proportion of Ki-67^+^ CD8^+^ T cells, together with reduced expression of exhaustion-associated markers and enhanced cytokine secretion by CD8^+^ T cells (Figures 8D–G). Furthermore, after treatment, the Ctcf-KD group exhibited greater sensitivity to therapy and higher tumor suppression rates compared to the Scramble group (Figures 8B,C), along with more pronounced activation of the immune microenvironment (Figures 8D–G). We further quantified myeloid-derived suppressor cells (MDSCs), macrophages, and dendritic cells (DCs) by flow cytometry (Supplementary Figures S3A,8H–J). The proportions of these immune cell populations remained unchanged upon Ctcf depletion. These findings suggest that CTCF expression promotes tumor growth and immune suppression in immunocompetent tumor-bearing mice, inducing resistance to chemo-immunotherapy.

**FIGURE 8:**
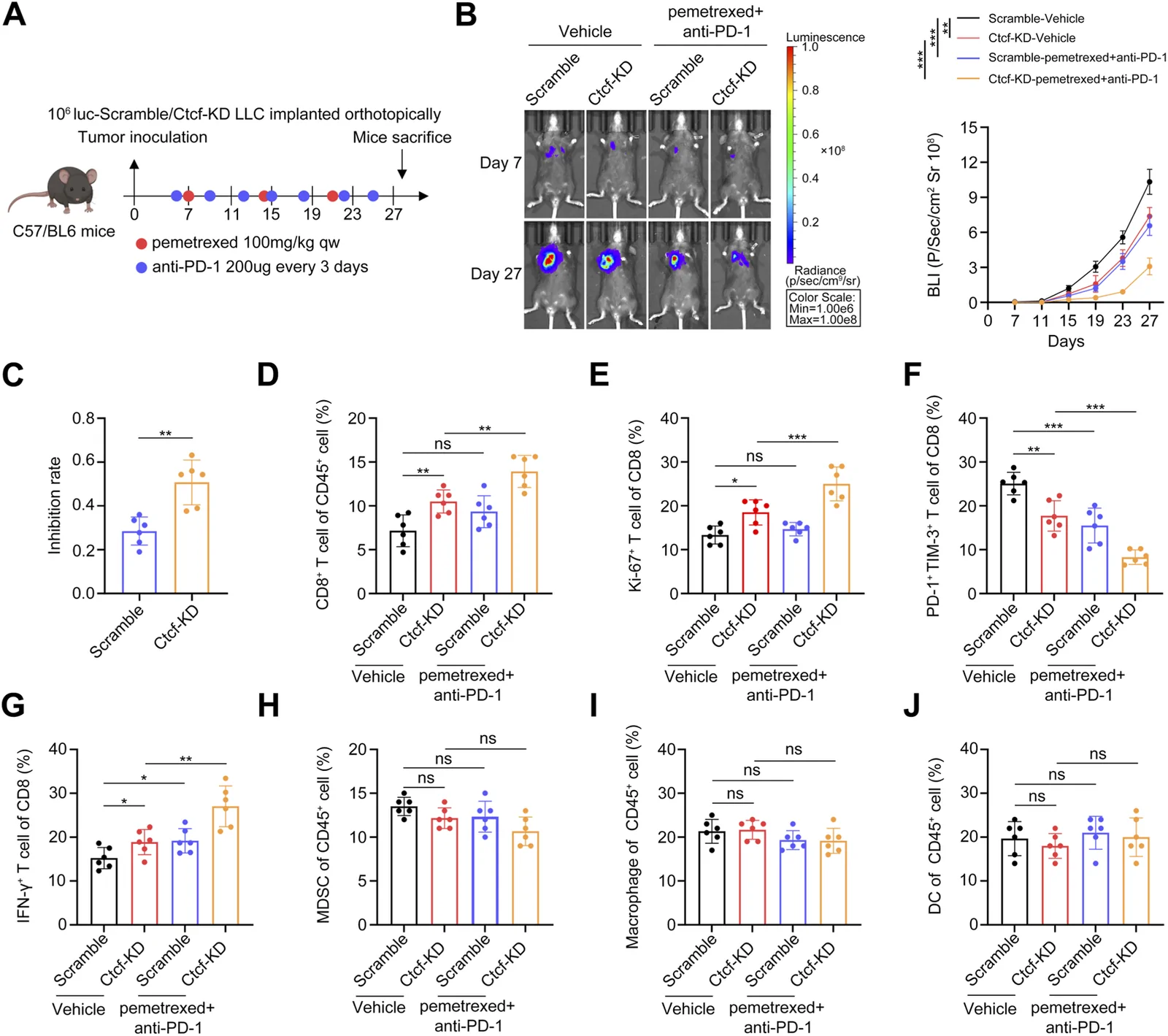
Downregulation of CTCF reverses chemo-immunotherapy resistance *in vivo*. **(A)** Schematic diagram of the *in vivo* experimental workflow. A total of 1 × 10^6^ luciferase-labeled LLC-Scramble or LLC-Ctcf-KD cells were orthotopically injected into the lungs of C57BL/6 mice (n = 6 per group). Mice were allocated to four experimental groups defined by tumor-cell genotype (Scramble or Ctcf-KD) and treatment (vehicle or pemetrexed plus anti-PD-1). Treatment began on day 4 after inoculation, tumor progression was monitored by *in vivo* bioluminescence imaging, and the experiment ended on day 28. **(B)** Representative bioluminescence images of each group on days 7 and 27 (left) and longitudinal quantification of tumor bioluminescence (right). **(C)** Effect of Ctcf knockdown on tumor inhibition rate. Tumor inhibition rate = (fluorescence intensity in the vehicle group − fluorescence intensity in the treatment group)/fluorescence intensity in the vehicle group × 100%. **(D–J)** Effect of Ctcf knockdown on immune cell infiltration in the tumor microenvironment of each group. **(D)** Proportion of infiltrating CD8^+^ T cells; **(E)** Proportion of Ki-67^+^ proliferating CD8^+^ T cells; **(F)** Proportion of PD-1^+^TIM-3^+^ exhausted CD8^+^ T cells; **(G)** Proportion of IFN-γ^+^ activated CD8^+^ T cells; **(H)** proportion of CD11b^+^Gr-1^+^ myeloid-derived suppressor cells (MDSCs); **(I)** proportion of CD11b^+^F4/80^+^ macrophages; and **(J)** proportion of CD11c^+^ dendritic cells (DCs). Unpaired two-tailed *t* tests were used for comparisons in **(B–J)**. Data are presented as mean ± SD. *, *P* < 0.05; **, *P* < 0.01; ***, *P* < 0.001; ****, *P* < 0.0001.

### 3.6 RRM2 and CD276 are key downstream effector molecules through which CTCF exerts its chemoresistance and immunosuppressive functions

We next investigated how CTCF promotes therapeutic resistance in lung adenocarcinoma cells. We established CTCF-OE and control cell lines in the human LUAD cell line A549 and performed bulk RNA sequencing. We analyzed the upregulated differentially expressed genes in CTCF-OE cells (fold change ≥2) for their associations with patient survival and CTCF expression in the TCGA-LUAD dataset (negatively correlated with survival and positively correlated with CTCF expression, correlation coefficient >0.3). These genes were further intersected with drug resistance–and immune-related gene sets, leading to the identification of two potential downstream effector genes of CTCF: RRM2 and CD276 (Figure 9A).

**FIGURE 9:**
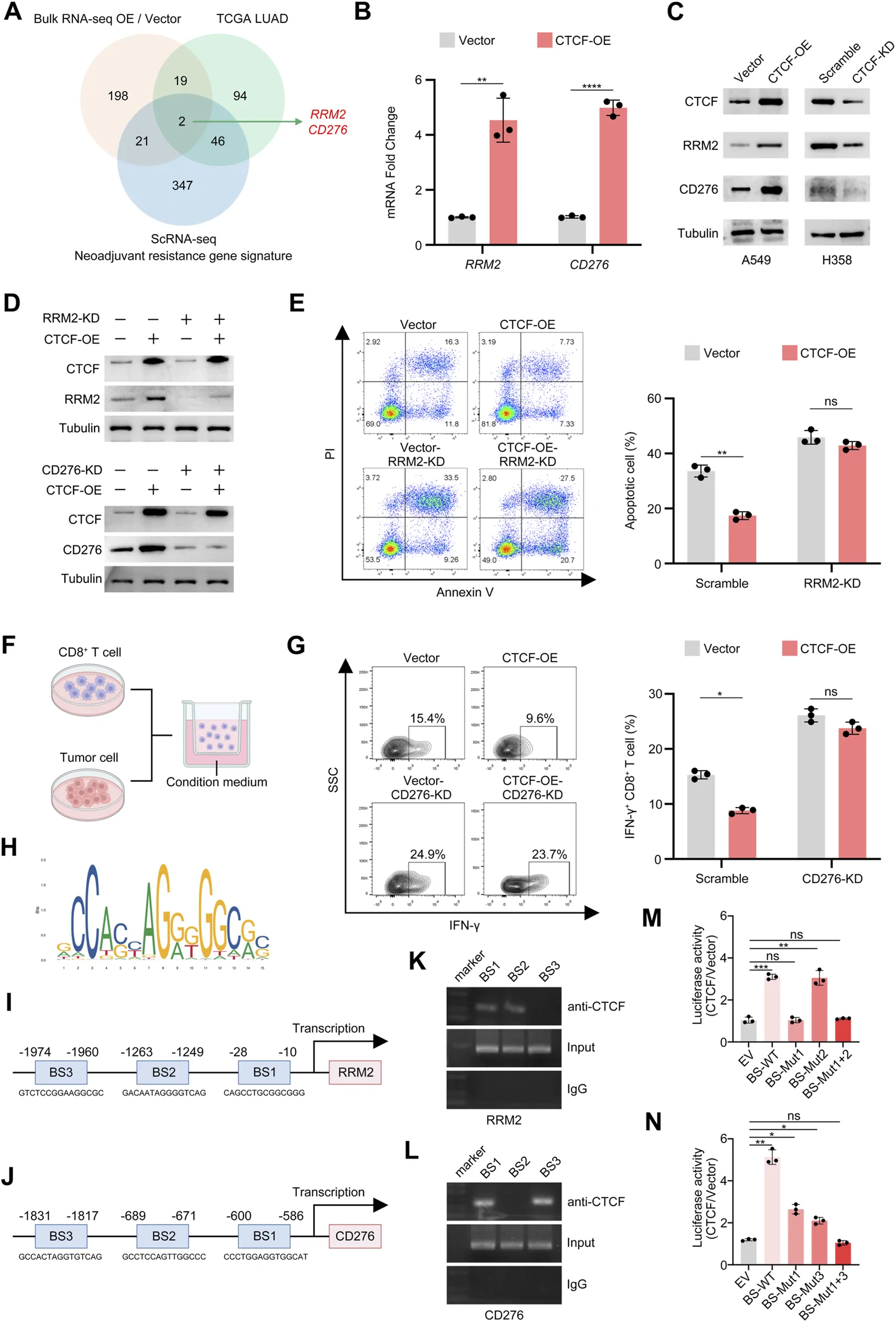
CTCF transcriptionally regulates RRM2 and CD276 to drive chemoresistance and immunosuppression. **(A)** The Venn diagram illustrates the strategy for screening candidate genes transcriptionally regulated by CTCF that are associated with chemoresistance and immunosuppression. **(B)** qPCR analysis of the effect of CTCF overexpression on RRM2 and CD276 mRNA levels in A549 cells. **(C)** Western blot analysis of the effect of CTCF overexpression and knockdown on RRM2 and CD276 protein levels in A549 cells. **(D)** Western blot analysis of the knockdown efficiency of RRM2 and CD276 in Vector/CTCF-OE A549 cells. **(E)** Effect of RRM2 knockdown on apoptosis of Vector/CTCF-OE A549 cells after pemetrexed treatment. **(F)** Schematic diagram of the experimental workflow: CD8^+^ T cells were treated with conditioned media from different tumor cells for 48 h, and their functional changes were detected. **(G)** Effect of CD276 knockdown on the ability of Vector/CTCF-OE A549 cells to inhibit the secretory activity of CD8^+^ T cells. **(H)** Schematic representation of the predicted CTCF binding motifs within the promoter regions of human RRM2 and CD276 genes. **(I)** Schematic representation of the three predicted CTCF-binding sites within the human RRM2 promoter. **(J)** Schematic representation of the three predicted CTCF-binding sites within the human CD276 promoter. **(K)** ChIP-qPCR analysis of the binding of CTCF to the three potential binding sites on the RRM2 promoters in the indicated A549 cell lines (n = 3 independent experiments). **(L)** ChIP-qPCR analysis of the binding of CTCF to the three potential binding sites on the CD276 promoters in the indicated A549 cell lines (n = 3 independent experiments). **(M)** Dual-luciferase reporter assay evaluating the effect of CTCF overexpression on RRM2 promoter activity in A549 cells. **(N)** Dual-luciferase reporter assay evaluating the effect of CTCF overexpression on CD276 promoter activity in A549 cells. Paired two-tailed *t* tests were used for comparisons in **(B,E,G,M,N)**. Data in these panels are presented as mean ± SD. *, *P* < 0.05; **, *P* < 0.01; ***, *P* < 0.001; ****, *P* < 0.0001.

RRM2 is the catalytic subunit of ribonucleotide reductase and serves as the rate-limiting enzyme for intracellular deoxyribonucleotide synthesis. Its overexpression has been shown to be closely associated with resistance to several antimetabolite chemotherapeutic agents, including gemcitabine and fluorouracil, in multiple cancer types. CD276 (also known as B7-H3) is an important immune checkpoint molecule of the B7 family that plays a critical role in tumor immune evasion by suppressing T-cell proliferation and cytokine secretion. We validated the regulatory effects of CTCF on RRM2 and CD276 expression at both the mRNA and protein levels (Figures 9B,C).

To further determine whether these two genes function as downstream mediators of CTCF, we knocked down RRM2 and CD276 in both Vector and CTCF-OE cells and examined the rescue of downstream phenotypes (Figure 9D). RRM2 knockdown significantly abrogated CTCF-mediated pemetrexed resistance *in vitro*, as evidenced by a marked increase in the proportion of apoptotic cells (Figure 9E). For CD276 knockdown experiments, conditioned media from the four groups of cells were collected and used to culture CD8^+^ T cells, after which their secretory functions were assessed. The results showed that CD276 knockdown significantly restored the suppression of IFN-γ secretion by CD8^+^ T cells induced by conditioned media from CTCF-OE cells (Figures 9F,G). Reciprocally, re-expression of RRM2 in CTCF-KD cells partially restored pemetrexed resistance, while CD276 re-expression partially reversed the enhanced IFN-γ secretion by CD8^+^ T cells induced by CTCF depletion (Supplementary Figures S4A–C). These findings support RRM2 and CD276 as functional downstream mediators of CTCF-dependent chemoresistance and immune suppression, respectively.

Subsequently, we examined how CTCF regulates RRM2 and CD276 transcription. Motif analysis identified three putative CTCF-binding sites within the −2-kb promoter regions of both genes (Figures 9H–J). ChIP assays showed CTCF occupancy at BS1 of the RRM2 promoter and at BS1 and BS3 of the CD276 promoter (Figures 9K,L). Consistently, dual-luciferase assays demonstrated that mutation of RRM2-BS1 markedly reduced CTCF-dependent promoter activation, whereas BS2 mutation had little additional effect (Figure 9M). For CD276, mutation of either BS1 or BS3 partially impaired promoter activation, while simultaneous mutation of both sites produced the strongest inhibition (Figure 9N). These results indicate that CTCF activates RRM2 primarily through BS1 and CD276 cooperatively through BS1 and BS3. Taken together, our results suggest that CTCF directly binds to the promoter regions of RRM2 and CD276, transcriptionally upregulates their expression, and thereby promotes chemotherapeutic resistance and immunosuppression in lung adenocarcinoma.

## 4 Discussion

In this study, we identified CTCF as a key transcriptional regulator associated with a chemo-immunotherapy resistance-related tumor cell state in lung adenocarcinoma. Through single-cell transcriptomic analysis, we first identified a malignant epithelial subpopulation enriched in patients with stable disease. By integrating public transcriptomic cohorts, machine-learning-based prognostic modeling, single-cell expression analyses, and survival validation, CTCF was further identified as a core candidate associated with high-risk status, tumor cell-intrinsic resistance, and poor clinical outcomes. Importantly, Ctcf knockdown significantly enhanced the antitumor efficacy of pemetrexed combined with anti-PD-1 treatment in an orthotopic lung tumor model and was accompanied by increased CD8^+^ T-cell infiltration, reduced T-cell exhaustion, and enhanced effector function. Mechanistically, CTCF directly bound to the promoter regions of RRM2 and CD276 and transcriptionally activated their expression, thereby promoting chemotherapy resistance and immune suppression, respectively. These findings suggest that the CTCF–RRM2/CD276 axis may represent an important mechanism linking tumor-cell-intrinsic chemoresistance with immune evasion.

Immune checkpoint inhibitors combined with chemotherapy have substantially changed the therapeutic landscape of NSCLC, particularly in the neoadjuvant and perioperative settings. The CheckMate 816 trial demonstrated that neoadjuvant nivolumab plus chemotherapy significantly improved pathological complete response and event-free survival compared with chemotherapy alone in resectable NSCLC (Forde et al., 2025). Similarly, the NADIM study supported the clinical activity of neoadjuvant chemotherapy plus nivolumab in locally advanced NSCLC (Mitsudomi et al., 2024). More recently, KEYNOTE-671 and CheckMate 77T further extended the role of PD-1 blockade to perioperative treatment and demonstrated improved clinical outcomes in early-stage or resectable NSCLC (Wakelee et al., 2023; Casc et al., 2024). Nevertheless, a considerable proportion of patients fail to achieve major pathological response or durable clinical benefit, indicating that treatment resistance remains a major obstacle to improving patient outcomes.

Previous studies investigating resistance to immunotherapy have mainly focused on the tumor immune microenvironment, including T-cell exhaustion, impaired antigen presentation, interferon signaling defects, and immunosuppressive stromal or myeloid populations (Sharma et al., 2017; Binnewies et al., 2018). Acquired resistance to immune checkpoint blockade has been associated with defects in antigen presentation and interferon pathway signaling (Zaretsky et al., 2016; Gettinger et al., 2017). Single-cell studies have further demonstrated that clonal revival and expansion of precursor exhausted T cells are closely associated with anti-PD-1 responses in lung cancer (Liu et al., 2022). Likewise, neoantigen-specific T-cell transcriptional programs have been linked to clinical responses to anti-PD-1 therapy (C et al., 2021). In contrast to these immune-centered studies, our work highlights the importance of tumor-cell-intrinsic transcriptional states as potential upstream drivers of both therapeutic resistance and immune suppression.

Recent multi-omic studies have increasingly emphasized the role of malignant cell states in determining therapeutic outcomes. Yan et al. integrated single-cell RNA sequencing, spatial transcriptomics, and bulk transcriptomic analyses and reported that resistance to chemo-immunotherapy was associated with distinct tumor cell states, COL11A1^+^ cancer-associated fibroblasts, SPP1^+^ macrophages, and tertiary lymphoid structure maturation status (Yan et al., 2025). Similarly, Barkley et al. demonstrated that recurrent cancer cell states exist across multiple tumor types and form specific interactions with the tumor microenvironment that contribute to immune evasion, metastasis, and therapeutic resistance (Barkley et al., 2022). Consistent with these observations, we identified a malignant epithelial cell subpopulation enriched in patients with poor responses to chemo-immunotherapy. Marker genes derived from this subpopulation were associated with metabolic reprogramming, proteasome activity, HIF-1 signaling, and poor prognosis, supporting the existence of a biologically aggressive and treatment-resistant tumor cell state.

CTCF is a multifunctional chromatin architectural protein that organizes three-dimensional genome structure, maintains local insulation, and contributes to transcriptional regulation (Ong and Corces, 2014; Nora et al., 2017; Hansen et al., 2019). Cancer-associated changes in CTCF occupancy and chromatin interactions can reshape oncogenic transcriptional programs (Fang et al., 2020). Previous work has therefore established broad roles for CTCF in tumor biology, while RRM2-mediated drug resistance and CD276-mediated immunosuppression have each been investigated in other settings (Ma et al., 2020; Picarda et al., 2016; Kontos et al., 2021; Zhou and Jin, 2021). However, these prior observations did not establish whether CTCF coordinates these two resistance programs within a chemo-immunotherapy-associated malignant cell state in lung adenocarcinoma.

Importantly, our findings suggest that CTCF simultaneously regulates tumor-cell sensitivity to therapy and the immune microenvironment. Ctcf knockdown significantly enhanced the efficacy of pemetrexed combined with anti-PD-1 treatment and promoted CD8^+^ T-cell infiltration and function *in vivo*. These findings are consistent with the concept that tumor-cell-intrinsic transcriptional programs can drive immune exclusion and resistance to checkpoint blockade (Jerby-Arnon et al., 2018). Our results further identify CTCF as an upstream regulator capable of coordinating both chemoresistance and immune suppression.

Mechanistically, we identified RRM2 as a critical downstream mediator of CTCF-induced chemotherapy resistance. RRM2 is an essential catalytic subunit of ribonucleotide reductase and plays a key role in deoxyribonucleotide synthesis, DNA replication, and DNA repair. High RRM2 expression has been associated with poor prognosis in lung adenocarcinoma (Ma et al., 2020). In our study, CTCF overexpression significantly increased RRM2 expression, whereas RRM2 silencing restored sensitivity to pemetrexed-induced apoptosis. Given that pemetrexed primarily exerts its antitumor effects through inhibition of folate metabolism and nucleotide synthesis, elevated RRM2 expression may provide tumor cells with a survival advantage by maintaining nucleotide pools and supporting DNA repair.

We also identified CD276 (B7-H3) as a key mediator of the immunosuppressive effects of CTCF. CD276 is a member of the B7 family and is highly expressed in many solid tumors while exhibiting limited expression in normal tissues. Increasing evidence suggests that CD276 represents an attractive target for cancer immunotherapy (Picarda et al., 2016; Kontos et al., 2021; Zhou and Jin, 2021). In the present study, CTCF overexpression increased CD276 expression and suppressed IFN-γ production by CD8^+^ T cells. Importantly, CD276 knockdown reversed this suppressive effect, indicating that CD276 is an essential downstream mediator of CTCF-dependent immune evasion.

Notably, ChIP-qPCR analyses confirmed direct binding of CTCF to the promoter regions of both RRM2 and CD276, elevating the relationship between these genes from simple expression correlation to direct transcriptional regulation. These findings support a dual-branch model in which CTCF promotes resistance to pemetrexed through RRM2 while simultaneously suppressing antitumor immunity through CD276. This integrated mechanism provides a plausible explanation for how a single transcriptional regulator can coordinate both chemoresistance and immune escape.

The core incremental contribution of this study is therefore not the isolated identification of CTCF, RRM2, or CD276 as cancer-associated factors. Rather, it lies in three connected findings: first, CTCF was nominated from a malignant epithelial state enriched in patients with suboptimal response to chemo-immunotherapy through an integrated single-cell and multi-cohort workflow; second, its functional relevance was tested in the context of pemetrexed plus anti-PD-1 treatment in an orthotopic model; and third, bidirectional perturbation and promoter-binding experiments linked CTCF to two distinct downstream branches, RRM2-associated pemetrexed resistance and CD276-associated suppression of CD8^+^ T-cell function. This study thus provides a context-specific integrative model connecting tumor-cell-intrinsic drug resistance with immune evasion, while not implying that the individual components or their broader oncogenic roles are newly discovered.

The prognostic model and immune microenvironment analyses further support the clinical significance of this resistance-associated state. The RSF + SuperPC model constructed from resistance-associated genes effectively stratified patient prognosis across TCGA and multiple GEO cohorts, suggesting that the transcriptional features of this tumor cell state are reproducible across independent datasets. High-risk tumors exhibited increased tumor purity but reduced immune and stromal scores. The ESTIMATE algorithm was originally developed to infer tumor purity and immune or stromal cell admixture from transcriptomic data (Yoshihara et al., 2013). Consistent results were obtained using multiple immune deconvolution algorithms, including TIMER, CIBERSORT, MCP-counter, xCell, and EPIC (Li et al., 2020; Newman et al., 2015). These findings suggest that CTCF-associated high-risk tumors may represent an immune-low and tumor-cell-dominant ecosystem.

Furthermore, increasing evidence indicates that B cells and tertiary lymphoid structures contribute to effective antitumor immunity and immunotherapy responses. Several studies have demonstrated that tertiary lymphoid structures are associated with improved clinical outcomes and responses to immune checkpoint blockade (Booth et al., 2026; Li et al., 2025; Petitprez et al., 2020). Mature tertiary lymphoid structures have also been reported to predict immune checkpoint inhibitor efficacy independently of PD-L1 expression (Vanhersecke et al., 2021). Therefore, the reduced immune infiltration associated with CTCF-high tumors may reflect impaired formation of effective antitumor immune niches.

From a translational perspective, these findings should be interpreted cautiously. CTCF may warrant evaluation as a biomarker of a resistance-associated tumor cell state, and the CTCF–RRM2/CD276 relationship provides a testable mechanistic framework linking chemotherapy resistance with immune suppression. Direct pharmacological inhibition of CTCF is not presently established and poses substantial challenges because CTCF is a broadly expressed, multifunctional genome organizer with essential and context-dependent functions, rather than a conventional enzyme with a well-defined drug-binding pocket. Systemic perturbation could therefore disrupt normal chromatin organization and transcription, creating a narrow therapeutic window and potential toxicity. Consequently, our data do not support immediate clinical targeting of CTCF. A more tractable hypothesis is to evaluate downstream vulnerabilities such as RRM2 or CD276, for which therapeutic strategies are under development (Kontos et al., 2021; Getu et al., 2023; Corrigan et al., 2025); however, whether such strategies overcome chemo-immunotherapy resistance specifically in CTCF-high lung adenocarcinoma requires pharmacological studies, biomarker-defined models, and prospective clinical validation.

Several limitations should be acknowledged. First, the single-cell cohort size was relatively limited and requires validation in larger independent cohorts. Second, the public validation datasets were primarily derived from bulk transcriptomic analyses rather than true chemo-immunotherapy-treated cohorts. Third, although direct transcriptional regulation of RRM2 and CD276 was confirmed, the broader chromatin regulatory network controlled by CTCF remains incompletely understood. Future studies incorporating CUT&Tag, ChIP-seq, ATAC-seq, and Hi-C analyses may provide a more comprehensive understanding of CTCF-mediated resistance programs. Fourth, the study establishes genetic and mechanistic associations but does not demonstrate the druggability, selectivity, or safety of CTCF inhibition; these issues require dedicated pharmacological and toxicological evaluation. Finally, the receptor and downstream signaling pathways responsible for CD276-mediated suppression of CD8^+^ T-cell function remain incompletely defined and warrant further investigation.

In conclusion, our study identifies CTCF as a key regulator of a chemo-immunotherapy resistance-associated tumor cell state in lung adenocarcinoma. Through direct transcriptional activation of RRM2 and CD276, CTCF promotes chemotherapy resistance and immune suppression, respectively. The CTCF–RRM2/CD276 axis therefore provides a mechanistic link between tumor-cell-intrinsic resistance and immune evasion and warrants further evaluation as a candidate biomarker and as a framework for investigating downstream therapeutic vulnerabilities, rather than as evidence that CTCF itself is currently a clinically actionable target.

## 5 Conclusion

This study identifies a chemo-immunotherapy resistance-associated malignant epithelial cell state in lung adenocarcinoma and demonstrates that CTCF is a key regulator of this resistant phenotype. By integrating single-cell transcriptomic analysis, multi-cohort validation, immune microenvironment assessment, and functional experiments, we show that high CTCF expression is associated with poor prognosis, immune exclusion, and reduced therapeutic sensitivity.

Mechanistically, CTCF directly activates RRM2 and CD276, thereby linking tumor-intrinsic chemoresistance with immune suppression. Ctcf knockdown enhances the antitumor efficacy of pemetrexed combined with anti-PD-1 therapy and restores CD8^+^ T-cell activity *in vivo*. These findings support further validation of the CTCF–RRM2/CD276 axis as a candidate biomarker and motivate investigation of its downstream vulnerabilities; they do not establish direct CTCF inhibition as a clinically feasible strategy.

## Data Availability

The datasets presented in this study can be found in online repositories. The names of the repository/repositories and accession number(s) can be found in the article/Supplementary Material.

## Glossary

ANOVA: Analysis of variance
ATAC-seq: Assay for transposase-accessible chromatin using sequencing
AUC: Area under the curve
CAF: Cancer-associated fibroblast
CD276: Cluster of differentiation 276 (B7-H3)
ChIP-qPCR: Chromatin immunoprecipitation quantitative polymerase chain reaction
ChIP-seq: Chromatin immunoprecipitation sequencing
CI: Confidence interval
CNV: Copy number variation
CR: Complete response
CTCF: CCCTC-binding factor
CTLA-4: Cytotoxic T-lymphocyte-associated protein 4
CUT&Tag: Cleavage under targets and tagmentation
DNA: Deoxyribonucleic acid
EMT: Epithelial-mesenchymal transition
EPIC: Estimating the Proportions of Immune and Cancer cells
ESTIMATE: Estimation of STromal and Immune cells in MAlignant Tumours using Expression data
GBM: Gradient boosting machine
GEO: Gene Expression Omnibus
GO: Gene Ontology
GSVA: Gene set variation analysis
GTEx: Genotype-Tissue Expression
Hi-C: Genome-wide chromosome conformation capture
HR: Hazard ratio
ICI: Immune checkpoint inhibitor
IFN-γ: Interferon-gamma
IPS: Immunophenoscore
KEGG: Kyoto Encyclopedia of Genes and Genomes
LLC: Lewis lung carcinoma
LUAD: Lung adenocarcinoma
MCP-counter: Microenvironment Cell Populations-counter
MDSC: Myeloid-derived suppressor cell
NK cell: Natural killer cell
NSCLC: Non-small cell lung cancer
PCA: Principal component analysis
PD-1: Programmed cell death protein 1
PD-L1: Programmed death-ligand 1
PR: Partial response
qRT-PCR: Quantitative real-time polymerase chain reaction
RECIST: Response Evaluation Criteria in Solid Tumors
ROC: Receiver operating characteristic
RRM2: Ribonucleotide reductase regulatory subunit M2
RSF: Random survival forest
scRNA-seq: Single-cell RNA sequencing
SD: Stable disease
SuperPC: Supervised principal components
TCGA: The Cancer Genome Atlas
TCIA: The Cancer Immunome Atlas
TIL: Tumor-infiltrating lymphocyte
TIME: Tumor immune microenvironment
TLS: Tertiary lymphoid structure
TME: Tumor microenvironment
t-SNE: t-distributed stochastic neighbor embedding
UMAP: Uniform Manifold Approximation and Projection
