# Supplementary material for: Integrated single-cell and multi-cohort analyses reveal CTCF as a key regulator of chemo-immunotherapy resistance in lung adenocarcinoma
Source: Front Cell Dev Biol. 2026 Sep 21;14:1922944. doi: 10.3389/fcell.2026.1922944 (PMC13635359; doi:10.3389/fcell.2026.1922944)
Supplement: Supplementary file 1 [file Table1.docx]

| **Supplementary Table 1** shRNA sequences for stable knockdown cell lines | |
| --- | --- |
| Name | Sequences (5′-3′) |
| H-CTCF shRNA1 (most effective) | AAAAGCCTCTTTCTTGGCAAAGTTTTTGGATCCAAAAACTTTGCCAAGAAAGAGGC |
| H-CTCF shRNA2 | AAAACCTCCTGAGGAATCACCTTAATTGGATCCAATTAAGGTGATTCCTCAGGAGG |
| H-CTCF shRNA3 | AAAAGCTCCTCAACACGATGGATAATTGGATCCAATTATCCATCGTGTTGAGGAGC |

| **Supplementary Table 2** Antibodies used for Western blotting | | | |
| --- | --- | --- | --- |
| Name | Brand | Catalog Number | Dilution Ratio |
| CTCF | CST | 2899S | 1:1000 |
| RRM2 | Abcam | ab172476 | 1:1000 |
| CD276 | Abcam | ab134161 | 1:1000 |
| Tubulin | STARTER | S0B0004 | 1:5000 |

| **Supplementary Table 3** Antibodies used for Flow cytometry | | | |
| --- | --- | --- | --- |
| Name | Brand | Catalog Number | Dilution Ratio |
| Fixable Viability Stain 780 | BD | 565388 | 1:1000 |
| Anti-Mouse CD45 | BD | 570291 | 1:100 |
| Anti-Mouse CD3 | BD | 564379 | 1:100 |
| Anti-Mouse CD8a | BD | 564422 | 1:100 |
| Anti-Mouse TIM-3 | BD | 568796 | 1:100 |
| Anti-Mouse PD-1 | BD | 562584 | 1:100 |
| Anti-Mouse CD11b | BD | 567469 | 1:100 |
| Anti-Mouse CD11c | BD | 565586 | 1:100 |
| Anti-Mouse F4/80 | BD | 569237 | 1:100 |
| Anti-Mouse Ly6g | BD | 740157 | 1:100 |
| Anti-Mouse Ly6c | BD | 568954 | 1:100 |
| Anti-Mouse IFN-γ | BD | 563376 | 1:100 |
| Annexin V-PE | YEASEN | 40302ES60 | 1:20 |
| Anti-Mouse Ki-67 | Biolegend | 652403 | 1:100 |

| **Supplementary Table 4** PCR primer information | |
| --- | --- |
| Name | Sequences (5′-3′) |
| Human ACTIN forward | CATGTACGTTGCTATCCAGGC |
| Human ACTIN reverse | CTCCTTAATGTCACGCACGAT |
| Human CTCF forward | GTGGAGCGATTAAACCGTGC |
| Human CTCF reverse | TTCTTTGGCTTCAGGTCCGT |
| Human RRM2 forward | ACAGAAGCCCGCTGTTTCTA |
| Human RRM2 reverse | GCCCAGTCTGCCTTCTTCTT |
| Human CD276 forward | TCCCTGAGTCCCAGAGTCG |
| Human CD276 reverse | GACGCAGCATCTTCCTGTGA |

| **Supplementary Table 5** ChIP primer information | |
| --- | --- |
| Name | Sequences (5′-3′) |
| Human RRM2 promoter CTCF binding site-1 forward | GAGCACCGAGGGAAAAAGGA |
| Human RRM2 promoter CTCF binding site-1 reverse | CCCAGGTCCGGCAACTTT |
| Human RRM2 promoter CTCF binding site-2 forward | CCAGCCACAGTTTGCAATTC |
| Human RRM2 promoter CTCF binding site-2 reverse | AAACTACGGGACATGGCGAG |
| Human RRM2 promoter CTCF binding site-3 forward | GGGGAACCGGGAGGTCCC |
| Human RRM2 promoter CTCF binding site-3 reverse | CATTGGCTGTGCCATGCCTC |
| Human CD276 promoter CTCF binding site-1 forward | CACAAGGCACAGGACTGAC |
| Human CD276 promoter CTCF binding site-1 reverse | CAGCAACCTGGGGAAGTAG |
| Human CD276 promoter CTCF binding site-2 forward | GCACGTAGACTCAAATAC |
| Human CD276 promoter CTCF binding site-2 reverse | CTATTTTGGCCAGGATGGTCTC |
| Human CD276 promoter CTCF binding site-3 forward | GCAAAATCTGGACAGGCC |
| Human CD276 promoter CTCF binding site-3 reverse | CTCTTTTCCTTCCACCCAC |
